# Supplementary material for: Microevolutionary hypothesis of the obesity epidemic
Source: PLoS One. 2024 Aug 7;19(8):e0305255. doi: 10.1371/journal.pone.0305255 (PMC11305523; doi:10.1371/journal.pone.0305255)
Supplement: S1 Text — (DOCX) [file pone.0305255.s001.docx]

**Supporting Information for**

Microevolutionary hypothesis of the obesity epidemic

Joseph Fraiman, MD^1,^*, Scott Baver^2^, PhD, Maciej Henneberg PhD, DSc, Dr h.c.^3^

***Corresponding Author:** Joseph Fraiman

Email: Josephfraiman@gmail.com

**S1 Fig.**  **Regressions of obesity rates per country in 1990 and in 2016 on the neonatal mortality in 1990.**

Please note the moderate but significant (p<0.001) negative relationship (correlation coefficients -0.45 - 0.48) -- the less neonatal mortality, the greater the obesity prevalence.

**S2 Fig.** **Relationship between the obesity prevalence rates, insufficient physical activity and caloric intake in countries with LMDR above the median.**


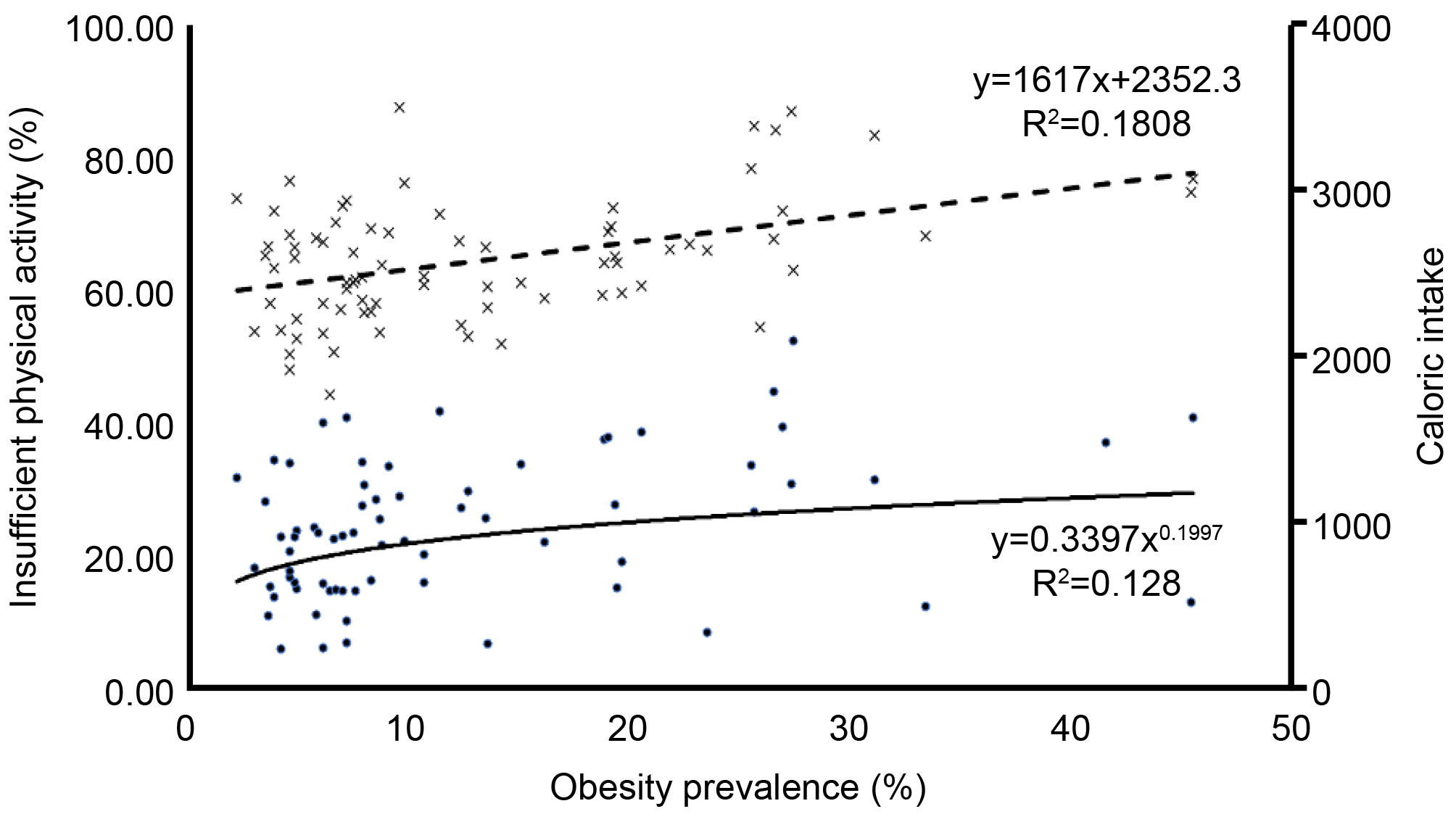


LMDR, Lifetime maternal death rate in 1990

Median LMDR=0.45%

**S1 Table.** **Partial correlation analysis between LMDR and obesity rates with other variables kept statistically constant.**

| Variables correlated | moment-product r | significance | N |
| --- | --- | --- | --- |
| Ln(LMDR 1990) |  |  |  |
| Ln(Obesity prevalence 2016) | -0.336 | p=0.00008 | 133 |

LMDR, Lifetime maternal death rate in 1990

Variables kept constant: insufficient physical activity, caloric intake, GDP and antibiotic consumption.

**S2 Table.** **Results of the stepwise multivariate regression analysis using the obesity rates as the dependent variable.**

| Variables correlated | Standard error | β coefficient | significance | R^2^ |
| --- | --- | --- | --- | --- |
| Ln(LMDR 1990) | -0.179 | 0.031 | <0.001 *** | 0.406 |
| Ln(Calorie intake 2017) | +0.789 | 0.395 | 0.047* | 0.423 |

LMDR, Lifetime maternal death rate in 1990

*p<0.05, *** p<0.001

**S3 Table. Partial correlation analysis between LMDR and obesity rates in countries with LMDRs above the median, with other variables are kept statistically constant.**

| Variables correlated | moment-product r | significance | N |
| --- | --- | --- | --- |
| Ln(LMDR 1990) |  |  |  |
| Ln(Obesity prevalence 2016) | -0.573 | p<0.00001 | 58 |

LMDR, Lifetime maternal death rate in 1990

Median LMDR=0.45%

Variables held constant: insufficient physical activity, caloric intake, GDP and antibiotic consumption

**S4 Table. Independent predictors of obesity prevalence in countries with LMDRs below the median**

| Variable | β | Std. Error | Sig. |
| --- | --- | --- | --- |
| Caloric intake | 1.173 | 0.519 | 0.027* |
| GDP | -0.078 | 0.106 | 0.465 |
| LMDR 1990 | -0.011 | 0.062 | 0.863 |
| Insuf.phys.activity | 0.370 | 0.184 | 0.048* |
| Antibiotic consumption | -0.066 | 0.137 | 0.632 |

LMDR, Lifetime maternal death rate in 1990; Insuf.phys.activity Insufficient physical activity

Median LMDR=0.45%

Based on multiple linear regression modelling using logarithmed variable values. Df1=5, df2=65. Adjusted R^2^ = 0.120.

A Pearson’s χ2 for the hypothesis that the rows and columns in a two-way table are independent accounting for correlations within families yields P > Χ2 = 0.1550, χ2(2) =1.88.

*p<0.05

**S1 File. Supplemental Analysis 1: FTO Allele Obesogenic Genotype Frequencies Pre and Post 1942**

FTO allele has been identified by GWAS studies to be associated with an increased risk of obesity, for both heterozygote (AT) and homozygote (AA), compared with homozygote (TT). The risk of obesity for AT was found to be an OR of 1.31 95% CI = 1.23 to 1.39), while for AA the OR was OR = 1.67; (95% CI = 1.47 to 1.89). With each additional allele adding 0.36 kg/m2, with homozygotes (AA) having higher BMI than heterozygotes (AT), and both having higher BMI than homozygote (TT) (1). The frequency of the obesity promoting FTO genotypes pre and post obstetrics were reported in Rosenquist *et al.* (2) using pre 1942 data and post 1942 data. The frequency of obesity promoting genotypes (AA/AT) were compared with obesity protective genotype (TT), prior to and after 1942. Rosenquist *et al.* (2) reported reported pre-1942 population frequency of obesity promoting genotypes (AA/AT) of 63.5% and obesity protective genotype (TT) of 36.5%. Post 1942 obesity promoting genotype AA/AT representing 66.9% of the population, while the obesity protecting genotype of TT represented 33.1%. This the change in frequency of the obesity promoting and protective genotypes pre 1942 and post 1942 is statistically significant (chi square X2=19.6232, P<0.00001).

**S1 References**

1. T. M. Frayling *et al.*, A common variant in the FTO gene is associated with body mass index and predisposes to childhood and adult obesity. *Science* **316**, 889–894 (2007).

2. J. N. Rosenquist *et al.*, Cohort of birth modifies the association between FTO genotype and BMI. *Proc. Natl. Acad. Sci. U. S. A.* **112**, 354–359 (2015).

**S2 File. Supplemental Analysis 2: Additional Maternal Mortality Analyses In Least Developed and Lower Income Nations**

Examining only the least developed nations included a total of 47 nations, of which only 6 had low MMR, and 41 had high MMR.  Of the least developed nations with low MMR three (50%) had high obesity, and of those with high MMR one (2.4%) had high obesity (Chi Squared=15.206 P<0.0001).

To ensure the results of these analysis were not mainly driven by differences in GDP between the developing nations a second exploratory analysis was performed only in nations of low and lower middle income. This analysis included 77 nations of which 36 were low income and 41 were lower middle income.  Of the low and lower middle income nations 31 had low MMR and 46 had high MMR.  High obesity was found in 17 (54.8%) nations with low MMR versus one (2.2%) nation with high MMR (Chi Squared=28.677 P<0.00001).
